# Multimodal Imaging in Idiopathic Neuroretinitis with Localized Choroidal Insufficiency: A Case Report

**DOI:** 10.3390/medicina57070697

**Published:** 2021-07-08

**Authors:** Junwoo Lee, Kiyoung Kim

**Affiliations:** Department of Ophthalmology, Kyung Hee University Hospital, Kyung Hee University, Seoul 02447, Korea; joonwool@hanmail.net

**Keywords:** idiopathic neuroretinitis, optic disc edema, Macular star, swept-source optical coherence tomography

## Abstract

Neuroretinitis is a rare clinical entity, characterized by optic nerve edema and star-shape hard exudate around fovea. The clinical features include acute unilateral visual loss, dyschromatopsia, relative afferent pupillary defect and visual field abnormalities. Increased vascular permeability of the optic disc is the main pathophysiology. As it is a not fully known clinical entity, diagnosis is challenging. In this case, we use multimodal imaging to reveal pathophysiology and anatomical change of early mild neuroretinitis. *Case presentation*: A 28-year-old healthy woman presented to the clinic with mild blurred vision in her left eye. After complete ophthalmic examination, outer retinal thickening of the temporal peripapillary area and optic disc edema were observed. Two days after diagnosis, the retinal edema and visual symptoms were aggravated. A hard exudate, maybe a part of macular star, was observed. Multimodal imaging including optical coherence tomography (OCT), swept-source OCT angiography (SS-OCTA), fluorescein angiography, and indocyanine green angiography visualized choroidal thinning and insufficient circulation beneath the outer retinal edema. Following steroid pulse therapy, the retinal edema and blurred vision were completely resolved. *Conclusions*: Multimodal imaging suggested that unilateral optic disc edema and early macular star help the diagnosis of neuroretinitis. In SS-OCTA, we found focal choroidal insufficiency. The focal insufficient choroidal circulation might be a contribution factor for idiopathic neuroretinitis. Multimodal imaging including SS-OCTA may be a valuable tool for detecting and monitoring disease progression.

## 1. Introduction

Idiopathic neuroretinitis is reported as a rare disease characterized by acute unilateral visual loss with disc edema and macular exudates [1]. It mainly occurs in the third and fourth decades of life, mostly unilateral [1,2]. It is self-limiting disorder, absence of recurrence and good visual prognosis [1,3]. The etiology is described by infectious and non-infectious cause. Due to its various etiology, differential diagnosis and careful evaluation is needed. In this case, we use multimodal imaging in early, mild neuroretinitis, to reveal choroidal vasculature and help the final diagnosis.

The present case shows multimodal imaging features of idiopathic neuroretinitis with swept-source optical coherence tomography angiography (SS-OCTA), which may therefore contribute to achieving a better understanding of the underlying pathophysiology of the disease.

## 2. Case Presentation

**Figure 1:**
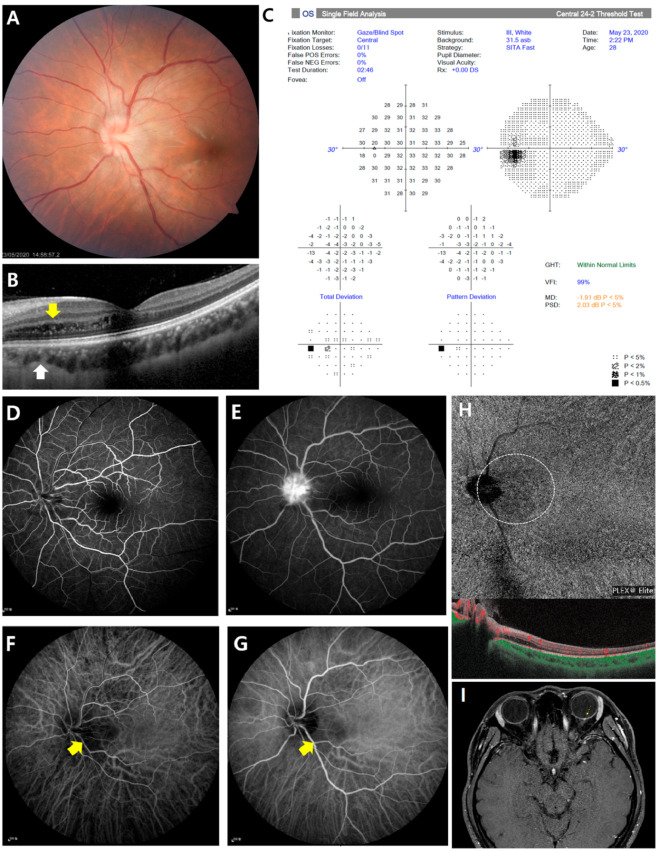
Initial multi-modal images from a 28-year-old woman with idiopathic neuroretinitis in her left eye. (**A**) Color fundus photo revealed mild optic disc swelling with blurred disc margin and lineally deposited hard exudates in the temporal peripapillary area. (**B**) Linear outer retinal thickening of with intraretinal cysts was observed in the nasal parafoveal area in spectral domain optical coherence tomography image. Corresponding enhanced depth imaging optical coherence tomography (OCT) images showed obvious thinning of the choroid beneath the intraretinal edema. (**C**) Visual field examination showed no abnormality. (**D**) Early-fluorescein angiography (FA) showed no arterial filling delay or non-perfusion area. (**E**) Mid-FA showed leakage at the optic disc without late staining of the venous walls. (**F**) Early-phase indocyanine green angiography (ICGA) images show focal hypofluorescent lesions in temporal peripapillary area. (**G**) Focal hypofluorescent area are more prominently visualized in the mid-phase ICGA. (**H**) Swept-source OCT angiography at the level of the choriocapillaris demonstrated focal vascular dropout beneath the intraretinal edema, which is located in the same area as the hypoperfusion in ICGA. (**I**) Orbital axial magnetic resonance imaging (MRI) shows tiny nodular enhancing lesion confined to the optic disc.

A 28-year-old woman consulted the ophthalmologist for vague blurring of vision in her left eye from the early morning. She had a history of dengue fever last year when she traveled to South America. Her medical history and ocular history were otherwise unremarkable. Best corrected visual acuity was 20/20 in her right eye and 20/32 in left eye. Anterior segment examination was unremarkable, and intraocular pressure was measured to be 21 mmHg in the right eye and 19 mmHg in the left eye. The ocular movement and pupil were normal without relative afferent pupillary defect (RAPD), but she complained of mild left ocular pain with eye movement. Color vision was normal with Ishihara’s plates in both eyes. Fundus examinations revealed mild optic disc edema and hyperemia in the left eye (Figure 1A). Spectral domain optical coherence tomography (SD-OCT) revealed intraretinal edema and hyperreflective spots in the outer nuclear layer of the temporal peripapillary area. Enhanced depth imaging OCT found thinning of the nasal parafoveal choroid beneath the intraretinal edema (Figure 1B). A Humphrey visual field 24-2 test showed no scotoma or defect in both eyes (Figure 1C). Systemic laboratory workup including serologic test, immunologic test, the complete blood count, serum C-reactive protein, erythrocyte sedimentation rate, D-dimer, lupus test, antinuclear antibody, and coagulation test were confirmed to rule out underlying conditions that might cause infectious disorder. Fluorescein angiography (FA) showed leakage at the disc with no evidence of retinal vasculitis or late staining of the venous walls (Figure 1D,E). Indocyanine green angiography (ICGA) showed focal hypofluorescence in the temporal peripapillary areas corresponding to intraretinal edema (Figure 1F,G). We additionally evaluated the perfusion status of the segmented retina and choroid layer with swept-source OCT angiography (SS-OCTA). The superficial and deep retinal plexus seemed almost undisturbed, but hyperperfused areas were noted in the choriocapillaris slab of the temporal peripapillary area (Figure 1H). Brain and orbital magnetic resonance imaging (MRI) were also normal without optic nerve enhancement (Figure 1I).

**Figure 2:**
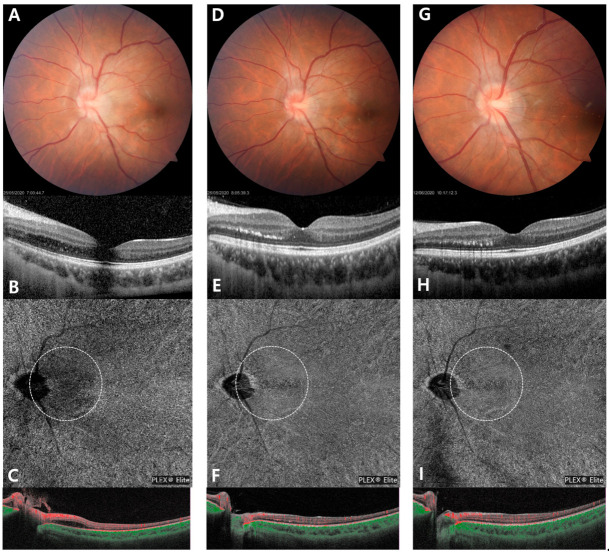
Follow-up multi-modal images at two days after the initial examination (**A**–**C**), two days following steroid pulse therapy (**D**–**F**), and one month after treatment (**G**–**I**). On the serial color fundus photograph, mild optic disc edema was resolved, but linearly deposited hard exudates remained in the temporal peripapillary area (**A**,**D**,**G**). Although intraretinal edema was slightly aggravated at the second day of admission (**B**), it was completely resolved two days after the steroid pulse therapy (**E**). Localized choroidal thinning was also recovered, but hyperreflective spots linearly remained in the outer retina even one month after treatment (**H**). On the serial swept-source OCT angiography images, peripapillary flow deficits of the choriocapillaris increased on the second day of admission (**C**). After starting steroid pulse therapy, choriocapillaris flow deficits significantly reduced (**F**), which was maintained at the one-month follow-up visit.

The patient was young, with no defined risk factor for retinal ischemia. Her visual field was normal, and there were no indicative findings of optic neuritis in the orbit MRI. In addition, normal-appearing fundus except mild optic disc edema and subretinal fluid, excluding other disease, gave the final impression of neuroretinitis. On the follow-up examination after two days, aggravation of the intraretinal edema and enlargement of the hypoperfusion area were observed on SD-OCT and SS-OCTA (Figure 2A–C). The patient was admitted with a diagnosis of neuroretinitis. After rule out infection by history taking and laboratory test, she was treated with intravenous prednisolone 1 mg/kg/day. Blurred vision began to resolve after 2 days of treatment and visual acuity improved to 20/20. Intraretinal edema and optic disc edema had resolved at 3 days after the treatment. Hard exudates and hyperreflective dots were seen in the resolved area of the intraretinal edema on SD-OCT. The hyperreflective dots aligned to a part of macular star, reinforcing the diagnosis of neuroretinitis. Recovery of focal flow deficits in peripapillary area was observed on SS-OCTA (Figure 2D–F). The choriocapillary flow deficit was seen by SS-OCTA, so we added aspirin tablet 100 mg/day. She was discharged on oral prednisolone tapering and aspirin. Improved visual acuity was maintained without recurrence of the retinal edema and choriocapillaris flow deficits at the one-month follow-up visit (Figure 2G–I).

## 3. Discussion and Conclusions

The disease entity of neuroretinitis is believed to closely resemble disorders of unilateral optic disc edema. Especially at an early stage, when the classical features may not always present, diagnosis is difficult. In this case, fundoscopy of neuroretinitis is described as unilateral optic disc edema, with subsequent formation of a macular star. In this case, absence of severe visual loss, vessel tortuosity, vascular leakage and altitudinal visual fields defect assist in excluding similar disease entities such as papillitis, papilledema, papillophlebitis and anterior ischemic optic neuropathy (AION) [4,5]. A possible linkage between them is that inflammation in the retinal veins near the optic nerve head caused the disruption of retinal and choroidal blood flow. In this case, both ICGA and SS-OCTA imaging showed focal choroidal insufficiency in the temporal peripapillary areas corresponding to hyperreflective intraretinal spots, which are a well-known OCT imaging biomarker of inflammation or damage of the optic disc vasculature.

Here, we detect focal choroidal circulation insufficiency in idiopathic neuroretinitis, using multimodal imaging. SS-OCTA can offer additional valuable insight into the current multimodal imaging techniques used for the characterization of neuroretinitis. Conventionally, FA and ICGA are used for the qualitative clinical assessment of retinal and choroidal circulations. ICGA allows more efficient visualization of the choroidal vasculature, since ICG shows less leakage from the choriocapillaris and has longer wavelengths for the retinal pigment epithelium transmission [6]. Hypofluorescence on ICGA can be seen as a result of nonperfusion of the choriocapillaris or impaired choroidal ICG diffusion [7]. In this case, hypofluorescence seen on ICGA probably represents the choriocapillaris changes secondary to retinal vascular inflammation. OCTA is a noninvasive and rapid imaging technique that can acquire volumetric angiographic scans segmented to specific depth of layers. The recently introduced SS-OCTA has improved imaging of the choroid due to its higher scanning rate and deeper penetration, which uses a longer wavelength. In these OCTA, choriocapillaris en face images; small dark regions called flow voids are more likely to occur secondary to choriocapillaris vascular dropout or flow reduction [8].

The fluid exudate and edema of the optic nerve fibers due to infectious process or inflammation is known as pathogenesis [4]. Depending on the cause, it is treated with steroid, antibiotics or combination therapy. Neuroretinitis is self-limiting disease. When symptoms are controlled, the visual prognosis is excellent and recurrence is rare. In this case, it was necessary to differentiate diagnosis resembling entities, due to vague symptom. After excluding infectious causes, treatment was started with steroid pulse therapy. The macular edema decreased and the macular star pattern exudate was confirmed.

The current case may be involved in the possible pathophysiology of neuroretinitis. Multimodal imaging demonstrated that the disturbance of choroidal flow corresponds to the localized peripapillary area involved by intraretinal edema. Esaki Y et al. [9] previously reported choroidal involvement in both posterior pole and mid-peripheral retina by OCT and ICGA imaging in idiopathic neuroretinitis, which supports the current findings as neurochorioretinitis. Intraretinal edema was resolved leaving a hard exudate, so-called macular star. We hypothesized that intraretinal thickening and hyperreflective spots are signs of peripapillary retinal venous inflammation, which causes focal choroidal flow deficit. Furthermore, widefield SS-OCTA can detect flow deficit areas at the level of choriocapillaris in the acute phase of neuroretinitis. Diminution of the peripapillary vasculature associated with disc edema, reduction of blood flow [10]. SS-OCTA also may be utilized in the noninvasive monitoring of retinal vascular disease progression or resolution based on microvascular assessment.
